# Alkylphenol Xenoestrogens with Varying Carbon Chain Lengths Differentially and Potently Activate Signaling and Functional Responses in GH_3_/B_6_/F10 Somatomammotropes

**DOI:** 10.1289/ehp.0800182

**Published:** 2008-12-31

**Authors:** Mikhail Y. Kochukov, Yow-Jiun Jeng, Cheryl S. Watson

**Affiliations:** Department of Biochemistry and Molecular Biology, University of Texas Medical Branch, Galveston, Texas, USA

**Keywords:** bisphenol A, calcium oscillation, ERK activation, estradiol, hydrophobicity, non-genomic response, prolactin release

## Abstract

**Background:**

Alkylphenols varying in their side-chain lengths [ethyl-, propyl-, octyl-, and nonylphenol (EP, PP, OP, and NP, respectively)] and bisphenol A (BPA) represent a large group of structurally related xenoestrogens that have endocrine-disruptive effects. Their rapid nongenomic effects that depend on structure for cell signaling and resulting functions are unknown.

**Objectives:**

We compared nongenomic estrogenic activities of alkylphenols with BPA and 17β-estradiol (E_2_) in membrane estrogen receptor-α–enriched GH_3_/B_6_/F10 pituitary tumor cells. These actions included calcium (Ca) signaling, prolactin (PRL) release, extracellular-regulated kinase (ERK) phosphorylation, and cell proliferation.

**Methods:**

We imaged Ca using fura-2, measured PRL release via radioimmunoassay, detected ERK phosphorylation by fixed cell immunoassay, and estimated cell number using the crystal violet assay.

**Results:**

All compounds caused increases in Ca oscillation frequency and intracellular Ca volume at 100 fM to 1 nM concentrations, although long-chain alkylphenols were most effective. All estrogens caused rapid PRL release at concentrations as low as 1 fM to 10 pM; the potency of EP, PP, and NP exceeded that of E_2_. All compounds at 1 nM produced similar increases in ERK phosphorylation, causing rapid peaks at 2.5–5 min, followed by inactivation and additional 60-min peaks (except for BPA). Dose–response patterns of ERK activation at 5 min were similar for E_2_, BPA, and PP, whereas EP caused larger effects. Only E_2_ and NP increased cell number. Some rapid estrogenic responses showed correlations with the hydrophobicity of estrogenic molecules; the more hydrophobic OP and NP were superior at Ca and cell proliferation responses, whereas the less hydrophobic EP and PP were better at ERK activations.

**Conclusions:**

Alkylphenols are potent estrogens in evoking these nongenomic responses contributing to complex functions; their hydrophobicity can largely predict these behaviors.

Variable–carbon-chain–length *para*-substituted alkylphenols and bisphenol A (BPA) are a structurally related group of xenoestrogens (Figure 1) that accumulate in the environment from the use of some detergents, paints, herbicides, pesticides, and plastic polymers. This includes BPA, 4-*n*-octylphenol (OP), and 4-*n*-nonylphenol (NP) (Tsai 2006; White et al. 1994). They can also biodegrade from phytoestrogens or hydrocarbon pollutants, such as 4-*n*-ethylphenol (EP) and 4-*n*-propylphenol (PP) (Burback and Perry 1993; Lundh et al. 1990). These chemicals frequently persist in air, soil, and aquatic environments in concentrations that can affect the reproductive health of animals, and probably humans (Ahel et al. 2000; Cincinelli et al. 2003, 2007; Clark et al. 1992; deJager et al. 1999a, 1999b; Dekant and Voelkel 2008; Han et al. 2004; Hossaini et al. 2001; Isobe et al. 2001; Moon et al. 2007; Routledge and Sumpter 1997; Tsai 2006; Ying et al. 2002). Although these compounds are best known for disrupting reproductive functions, they are increasingly found to affect nonreproductive functions regulated by estrogens, as well (Watson et al. 2007a). Alkylphenol xenoestrogens are becoming ever more environmentally ubiquitous, owing to their chemical stability and bioaccumulation; they are also stored in the body fat of animals and humans (Ahel et al. 1993; Calafat et al. 2005; Tan and Mohd 2003; Ye et al. 2006). Their importance as contaminants and the study of their biological effects are increasingly complicated by their further modification by chlorination in manufacturing and wastewater treatment plants (Fukazawa et al. 2001; Gallard et al. 2004; Gross et al. 2004; Hu et al. 2002; Petrovic et al. 2003).

Previous structure–activity relationship studies have shown that these compounds bind to the nuclear estrogen receptor-α (ER-α) with a *K_i_* of 900 nM to 600 mM. The receptor affinity of these alkylphenols increases with increasing chain length of the alkyl groups (Tabira et al. 1999). Their binding affinity to nuclear ER, as well as their ability to activate nuclear ER-α–mediated functions, is several orders of magnitude less potent than that of 17β-estradiol (E_2_) (Bonefeld-Jorgensen et al. 2007; Kwack et al. 2002; Routledge and Sumpter 1997). However, these parameters could be dramatically different for membrane ERs (mERs), which are still poorly characterized and function in a very different microenvironment.

The estrogenic actions of alkylphenol compounds are less studied compared with BPA (vom Saal and Hughes 2005; Welshons et al. 2006); however, several studies clearly demonstrated multiple effects of OP and NP, such as changes in gene transcription, cell proliferation, and organ development, in a variety of cell models (Bonefeld-Jorgensen et al. 2007; Soto et al. 1991; Sumpter and Jobling 1993, 1995; White et al. 1994) and animals (deJager et al. 1999a, 1999b; Hewstone 1994; Hossaini et al. 2001; Moon et al. 2007; Routledge and Sumpter 1997). These researchers noted that the estrogenic effects of alkylphenols are evident at relatively high (0.1–1 μM) concentrations. Unfortunately, many past studies have not explored lower doses. At the cellular level, little is known about rapid, mER-mediated estrogenic effects, which have recently been shown to have nonconventional dose–response relationships (Watson et al. 2007b), where the shape of the curve is nonmonotonic, with more than one dose–response peak of activation. Therefore, the magnitude of these measurable biologic responses does not appear to correlate with simple receptor occupancy, but two or more different subpopulations of the receptor that we do not yet understand (perhaps in different subcellular or submembrane locations) could explain this. Such anomalies could also be due to the complex, multiple-pathway and multistep regulation of cell-signaling events initiated by binding to receptors at the plasma membrane (Bulayeva et al. 2004).

In our previous study of nongenomic xenoestrogen responses in mER-α–enriched GH_3_/B_6_/F10 somatomammotropes, both BPA and NP activated calcium (Ca) signaling and prolactin (PRL) release, whereas only NP increased extracellular-regulated kinase (ERK) phosphorylation (Bulayeva and Watson 2004; Stroev et al. 2001; Wozniak et al. 2005) for these conditions/time points. These observations suggested that phenolic xenoestrogens, acting through mERs, could have potent effects on pituitary cell function via non-genomic mechanisms, but could also differ in some aspects of evoked responses. That low concentrations of these phenolic compounds with much lower affinity for nuclear ER-α still produced potent membrane-initiated signaling effects led us to inquire about their structure–activity relationships, which were likely to be different than those reported for nuclear ER-α. In this study, we assessed the nongenomic estrogenic activities of four different alkylphenols that vary in their side-chain lengths (2, 3, 8, and 9 carbons) and hydrophobicity, compared with BPA and E_2_ by testing their rapid effects on Ca signaling, ERK activation, cell proliferation, and PRL release in GH_3_/B_6_/F10 cells.

## Materials and Methods

### Materials and cell culture

We purchased paraformaldehyde and glutaraldehyde from Fisher Scientific (Pittsburgh, PA) and fura-2 acetoxymethyl ester from Molecular Probes (Eugene, OR). The following alkylphenols (purity, catalog no.) were purchased from Aldrich (St. Louis, MO): EP (99%, no. E44205), PP (99%, no. P53802), OP (99%, no. 384445), and NP (technical grade, no. 29,085-8). We purchased the phosphorylated ERK (pERK) antibody (Ab) used in the ERK phosphorylation studies from Cell Signaling Technology (Danvers, MA). All other materials were purchased from Sigma (St. Louis, MO).

We performed all experiments on the mER-α–enriched GH_3_/B_6_/F10 subclone (Pappas et al. 1995) of GH_3_/B_6_ pituitary somatomammotropes, which are known to secrete PRL in response to both thyrotropin-releasing hormone (TRH) and E_2_ (Dufy et al. 1979) and have electrophysiologic properties typical of anterior pituitary neuroendocrine cells (Dufy et al. 1979; Glassmeier et al. 2001). Cells were maintained at 5% CO_2_, 37°C in phenol red–free Dulbecco’s modified Eagle’s medium (DMEM; Mediatech, Herndon, VA) with 12.5% horse serum (Gibco BRL, Grand Island, NY) and 2.5% defined supplemented calf serum and 1.5% fetal bovine serum (Hyclone, Logan, UT).

Before experiments, we deprived cells of serum steroids by placing them in DMEM without serum, with or without defined additives, or with 4× charcoal-stripped serum (see individual experimental descriptions for type of medium and length of serum deprivation).

### Ca imaging experiments

Before Ca-imaging experiments, we harvested cells with 0.25% trypsin/0.02% EDTA, plated them on poly-D-lysine–treated 35/22-mm glass-bottom dishes (Willco Wells, Amsterdam, Netherlands) at a density of 100,000 cells/mm^3^, and incubated them at 37°C for 48–72 hr. At 12 hr before an experiment the medium was changed to serum-free, phenol red–free DMEM. On the day of the experiment, the cells were loaded with 2.5 μM fura-2 acetoxymethyl ester (Molecular Probes), a Ca-sensitive fluorescent dye (Grynkiewicz et al. 1985), for 1 hr at room temperature (RT), washed three times, and then maintained at RT for 1–4 hr before live Ca-imaging experiments. The physiologic solution used for fura-2 loading and live-cell imaging contained 150 mM NaCl, 5.5 mM KCl, 1 mM MgCl_2_, 4 mM CaCl_2_, 7 mM glucose, and 10 mM HEPES, pH 7.4. The cell imaging setup included a Nikon 200E microscope with 20× SuperFluo objective and a computer-controlled illumination system (Sutter Instruments, Novato, CA) equipped with a digital monochrome-cooled charge-coupled Roper Coolsnap HQ camera (Roper Scientific, Tucson, AZ). We acquired fluorescent emissions at 510 nm (from regions of interest corresponding to a single cell) online with MetaFluor software (Universal Imaging, Downington, PA) in dual 340/380-nm excitation mode, with the 340:380 ratios reflecting cytosolic Ca. We used PeakFit and SigmaPlot (Systat Software, Chicago, IL) and MetaMorph (Universal Imaging) software for conversion and analysis of the data.

### ERK phosphorylation assay

We previously developed a 96-well plate assay to assess activated ERK-1 and -2 levels in fixed cells (Bulayeva et al. 2004), which we used here to assess these structurally related alkylphenols. Cells were plated at a density of 10,000 cells/well. The next day, we replaced the growth media with DMEM containing 1% 4× charcoal-stripped serum for 48 hr. Cells were then washed with DMEM once before the estrogens (or 0.0001% ethanol vehicle control) were added for 5 min. We then fixed the cells with 2% paraformaldehyde/0.2% picric acid at 4°C for 48 hr, permeabilized them with PBS containing 2% bovine serum albumin (BSA) and 0.1% Triton X-100 for 1 hr at RT, and washed them three times with PBS before adding primary Ab against pERK (1:400 in PBS/1% BSA/0.1% Triton X-100). After overnight incubation at 4°C, the cells were washed three times with PBS and the biotin-conjugated secondary Ab (Vector Labs, Burlingame, CA; 1:300) in PBS/1% BSA was added for 1 hr at RT. The cells were again washed with PBS, incubated with Vectastain ABC-AP solution (Vector Labs) for 1 hr at RT, and again washed three times with PBS, followed by addition of Vectastain alkaline phosphatase substrate plus levamasole (an endogenous phosphatase inhibitor). We incubated plates in the dark for 30 min at 37°C and then read the signal for the phosphatase product *para*-nitrophenol (pNP) at 405-nm absorbance (A_405_) in a model 1420 Wallac microplate reader (Perkin Elmer, Waltham, MA). We determined the number of cells in each well by the crystal violet (CV) assay.

### PRL release measured via radioimmunoassay

Cells (0.5–0.7 × 10^6^) were plated in poly-D-lysine–coated six-well plates. After serum deprivation in DMEM containing 5 μg/mL insulin, 5 μg/mL transferrin, 5 ng/mL sodium selenite, and 0.1% BSA for 48 hr, we removed this medium and added new DMEM/0.1% BSA with or without the appropriate reagent or vehicle control (ethanol). The cells were incubated for 1 min and centrifuged at 4°C, 350 × *g* for 5 min. We then collected the supernatant and stored it at −20°C until radioimmunoassay (RIA) for PRL, and fixed the cells for cell number estimation with the CV assay (see below). Concentrations of PRL were determined using components of the rat PRL RIA kit from the National Institute of Diabetes and Digestive and Kidney Disease and the National Hormone and Pituitary Program (Baltimore, MD). Briefly, we combined 100 μL cold standard (rat PRL-RP-3) or unknown sample, 500 μL rPRL-s-9 antiserum [final dilution of 1:437,500 in RIA buffer containing 80% phosphate-buffered saline (PBS), 20% DMEM, and 2% normal rabbit serum], and 200 μL ^125^I-labeled rat PRL (Perkin Elmer, Wellesley, MA; using 15,000 counts/tube diluted in RIA buffer), which we incubated with shaking overnight at 4°C. Anti-rabbit IgG was added (200 μL of 1:9 final dilution in RIA buffer) and the samples were incubated with shaking at RT for 2 hr. We then added 1 mL polyethylene glycol (PEG) solution (1.2 M PEG, 50 mM Tris, pH 8.6) and incubated the samples with shaking at RT for 15 min. The samples were centrifuged at 4,000 × *g* for 10 min at 4°C, the supernatant decanted, and the pellet counted in a Wizard 1470 Gamma Counter (Perkin Elmer). We calculated the PRL concentration and normalized it to the CV values representing cell number.

### Cell number measurements: CV assay

We assessed cell numbers for proliferation studies, or normalization of the above assay results, by washing any other detection reagents or growth medium from the wells and then staining the cells with CV dye. CV stains multiple cellular constituents, with values proportional to cell numbers measured with other techniques (Zivadinovic et al. 2005). For proliferation studies, cells were plated at 5,000 cells/well onto poly-D-lysine–coated wells of a 96-well plate. The next day, we added growth media with 1% 4× charcoal-stripped serum with or without the different xenoestrogens or vehicle. After 72 hr, cells were fixed for 20 min in 2% paraformaldehyde and 0.1% glutaraldehyde in PBS, stained for 30 min with a 0.1% solution of CV, and destained in deionized water. The dye was released by 10% acetic acid at RT for 30 min and the A_590_ signal of the extract was read in a microplate reader.

### Statistics

We analyzed data from the measurements of PRL release, cell proliferation, and the pERK assay by one-way analysis of variance (ANOVA) followed by multiple comparisons versus the control group (Dunnett’s method). Paired *t*-tests were used to compare Ca signaling characteristics before and after various estrogen treatments, and an ANOVA with the Dunn’s method for multiple comparisons was used for the different treatment series. The SigmaStat 3.0 program (Systat Software, Chicago, IL) was used for all statistical analyses. We accepted significance at *p* < 0.05.

## Results

### Alkylphenol xenoestrogens activate cytosolic Ca oscillations

**Figure 2:**
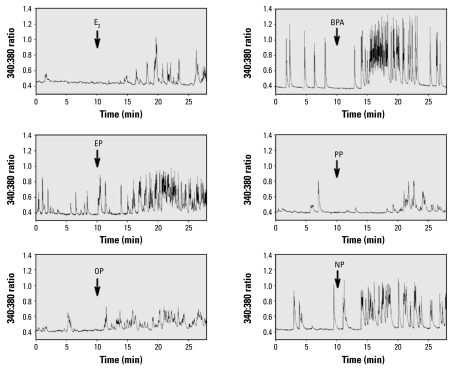
Rapid effect of E_2_, BPA, and alkylphenols on cytosolic Ca oscillations. Time-dependent Ca levels (340:380 ratio) were recorded from a single representative responding cell for each tested compound. We added each estrogen at the 10-min time point at a concentration of 1 nM, as indicated by the arrows.

Fura-2–loaded GH_3_/B_6_/F10 mER-α–enriched cells showed functional heterogeneity typical for this cell line (Bulayeva et al. 2005) and its parental cell line (Dufy et al. 1979; Glassmeier et al. 2001); 30–50% of simultaneously recorded cells generated some level of spontaneous Ca oscillations at a frequency from 0.1–10 spikes/min. To compare the changes in Ca responses caused by E_2_ and BPA with those caused by different alkylphenolic xenoestrogens, we treated cells with different concentrations of these compounds ranging from 1 fM to 1 nM. Figure 2 shows representative Ca traces recorded from single cells demonstrating significant increases in Ca oscillation frequencies after addition of 1 nM of each estrogen. As we observed previously in comparisons of E_2_ with estriol (E_3_) and estrone (E_1_) (Watson et al. 2008), there was a highly variable and relatively long (30 sec to several minute) delay in the response, but with no significant differences in this latency between treatment groups (data not shown). Responses to estrogens were typically observed in cells that already demonstrated some pretreatment level of spontaneous Ca firing (monitored during 10 min of recording); silent cells under these control conditions only rarely showed a response after treatment with an estrogen. Therefore, in subsequent analyses, we evaluated only cells that showed some spontaneous activity in prerecordings.

### Quantitation of different aspects of estrogen-induced Ca responses: dose responses and structure–activity relationships

**Figure 3:**
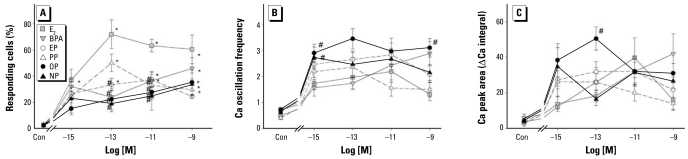
Quantitation of different aspects of estrogen-induced Ca responses. (*A*) The number of responding cells calculated as a percentage of total number of cells assessed in a given dish, recorded simultaneously (3–5 different dishes, 54–228 cells for each data point). (*B*) Changes in Ca oscillation frequency (average number of Ca peaks per minute, measured over 10 min) compared with the average baseline frequency before any estrogen treatment (Con). *n* = 11–33 individual cells for each point. (*C*) The integral of the Ca response to estrogens or vehicle-treated controls (Con), measured as the total area under the Ca peaks, recorded over 10 min. We subtracted the baseline pretreatment Ca levels. *p < 0.05 compared with vehicle control. #*p* < 0.05 compared with E_2_

Figure 3A quantifies the percentage of cells demonstrating increased Ca oscillation frequency in response to E_2_ or xenoestrogen treatment. E_2_ at concentrations from 1 fM to 1 nM increased the Ca firing rate in a significantly larger number of cells than did vehicle treatment, with the maximal effect at concentrations from 100 fM to 1 nM (where 60–70% of cells responded). As we found in an independent set of experiments, E_2_ increased average number of Ca-firing cells 30% per dish (data not shown; *p* < 0.05, *n* = 4).

The alkylphenol xenoestrogens were not quite as effective as E_2_ in eliciting a cell response; the lowest concentration that caused a significant increase as estimated by multiple comparison statistics was 100 fM for EP and PP, 10 pM for BPA, and 1 nM for OP and NP. However, *t*-test statistics showed a significant effect at concentrations as low as 1 fM for OP and BPA and 100 fM for OP and BPA; responses continued for these compounds at higher concentrations with some exceptions. The percentages of cells responding to these xenoestrogens were typically smaller than for E_2_ across all concentrations, rarely exceeding 40–50%. The magnitude of the response tended to increase with increasing concentrations of BPA and the long-chain alkylphenols OP and NP, but peaked and declined for the short-chain compounds EP and PP.

Figure 3, B and C, provides further evidence for differences in quantifiable Ca responses in GH_3_/G_6_/F10 cells to structurally different alkylphenols, compared with E_2_. We compared the dose dependence of estrogen-induced changes in Ca oscillation frequency, as well as the total magnitude of the Ca response (measured as the total area of the Ca spikes, or the ΔCa integral). We calculated these parameters only for responding cells over a 10-min interval. These dose–response curves were generated slightly differently from those reported in our previous study that included E_2_, NP, and BPA (Wozniak et al. 2005), as here each dose was analyzed with entirely independent treatments of different cell preparations, rather than multiple treatments of the same cells. Thus, we eliminated possible artifacts of delayed changes in cell responsiveness due to a prior treatment. Such effects can alter the number or functional state of plasma membrane receptors, or downstream effectors after repeated stimulation (desensitization). For both the Ca firing rates and the amounts of Ca getting into the cells, the long-chain alkylphenols OP and NP were more effective than E_2_ at many concentrations and largely comparable at others. Many of the responses (except to BPA) show a biphasic dose dependence (declining at the higher concentrations). They also appear in many cases to be all-or-nothing phenomena, as we saw previously for the other physiological estrogens, E_1_ and E_3_ (Watson et al. 2008). Overall, these studies indicate that the xenoestrogens not only were as potent and effective as E_2_, but in some cases evoked this response significantly better than E_2_, especially if the chain length was eight carbons.

### Rapid effects of alkylphenols on PRL release

**Figure 4:**
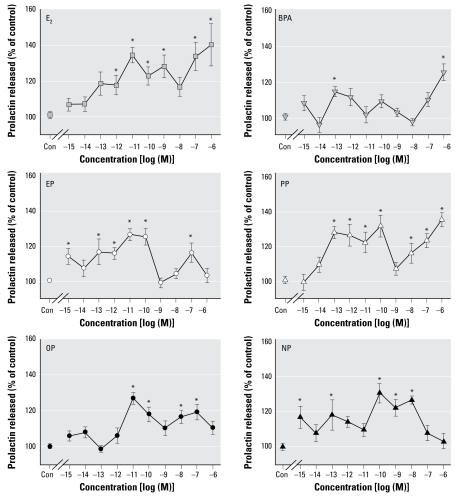
Concentration dependence of estrogen-induced rapid changes in PRL secretion. We measured PRL released into the medium by RIA after 1 min of treatment with E_2_, BPA, EP, PP, OP, or NP at different concentrations (*n* = 12–24 for each data point). *p < 0.05 versus vehicle control (Con; *n* = 32).

Because an increase in Ca firing in neuroendocrine cells activates peptide hormone secretion by priming and triggering secretory granule release, it would be reasonable to expect from the data shown in Figures 2 and 3 that the alkylphenols and BPA may have a rapid E_2_-like effect on PRL secretion by pituitary cells. As shown in Figure 4, E_2_ and the short-chain alkylphenols EP and PP showed very similar dose-dependent stimulating effects on PRL release by GH_3_/B_6_/F10 cells after a 1-min incubation at a concentration range from 100 fM to 100 pM. The overall characteristics of the responses were similar for all six compounds, with the responses disappearing in approximately the 1–10 nM range (even though for BPA and NP this was a trend and not strictly significant by the same tests). Both long- and short-chain alkylphenols were relatively potent in this response, with EP and NP causing responses at the lowest concentration (10^−15^ M). Therefore, differences here did not seem to correlate directly with the carbon chain length of the alkyl modification. This level of potency exceeds that of E_2_ by several orders of magnitude. Despite some differences in the most effective dose ranges, the magnitude of xenoestrogen- and E_2_-induced rapid PRL release was very similar.

### Alkylphenol xenoestrogens rapidly increase ERK phosphorylation

**Figure 5:**
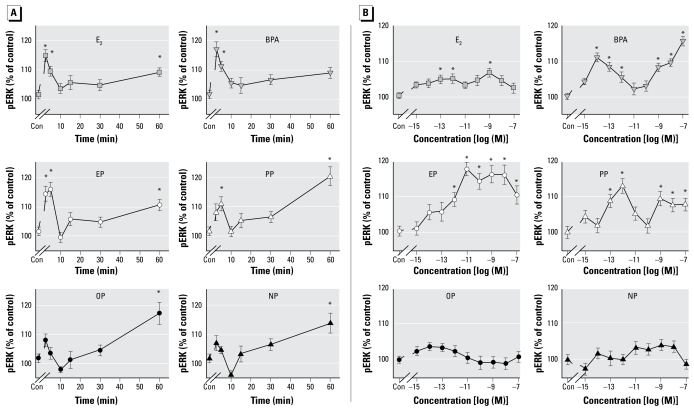
Changes in ERK phosphorylation in response to E_2_, BPA, EP, PP, OP, and NP. Values are the amount of pNP generated for each well, normalized to the CV value for cell number, presented as a percentage of vehicle-treated controls (Con), which we set to 100. (*A*) Time-dependent changes in the phosphorylation status of ERKs due to 1 nM estrogen treatments (*n* = 24 samples over three experiments). (*B*) Concentration-dependent changes in the phosphorylation status of ERKs after 5-min estrogen treatments (*n* = 32 samples over four experiments). *p < 0.05 versus control.

In a previous study, we demonstrated that E_2_ and several xenoestrogen compounds of different classes rapidly activate ERKs by phosphorylation in GH_3_/B_6_/F10 prolactinoma cells with distinct dose dependence and temporal activation patterns. Here, we compared the ability of a structurally related set of compounds to elicit this response. As shown in Figure 5A, most tested compounds produced characteristic oscillating increases in pERK with one rapid peak at 2.5–5 min (lasting only a few minutes), followed by a second slow, continuous increase reaching statistically significant levels in most cases at 60 min. Although short-chain alkylphenols, as well as E_2_ and BPA, produced comparable 10–15% increases in ERK phosphorylation at 2.5–5 min, the longer-chain compounds did not cause a statistically significant initial rise, so again, there were distinct differences based on chain length. BPA did not produce a significant second peak here, and also was not as effective as other compounds in our previous studies (Bulayeva and Watson 2004).

Figure 5B compares the dose dependence of the rapid ERK activation at 5 min. BPA and PP produced 10–20% increases in pERK with nonmonotonic dose–response characteristics. The magnitude of the effect exceeded that caused by E_2_, which showed a similarly shaped response curve. EP caused only a simple plateauing of the response curve, with no sensitivity to lower concentrations. However, the long-chained alkylphenols OP and NP caused no significant changes at this time point, in contrast to the significant NP dose–response changes at 30 min, which we reported previously (Bulayeva and Watson 2004). This result emphasizes the fact that we often see entirely different dose–response relationships at different time points for these oscillating responses. Overall, we again see a striking difference between the two chain-length subcategories of alkylphenols.

### Cell proliferation

**Figure 6:**
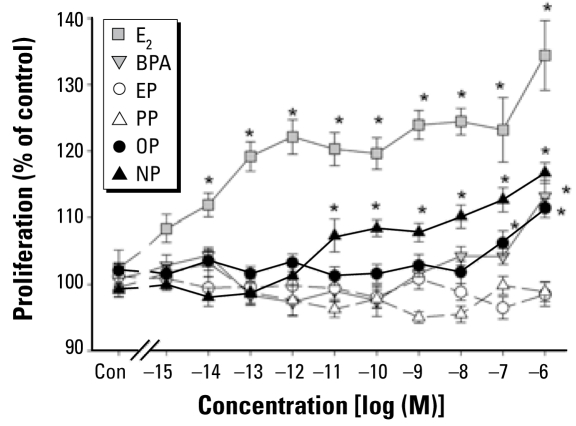
Proliferative response to E_2_, BPA, and alkylphenols measured by the CV assay. We grew cells in the presence of different concentrations of E_2_ or xenoestrogens for 3 days. Proliferation at day 3 is cell number as a percentage of vehicle control (Con). *n* = 24 samples over three experiments. *p < 0.05 versus control.

Despite the prominent effect that some compounds had on ERK phosphorylation, this did not correlate as expected with the effects on cell number (Figure 6). Although E_2_ had only a moderate effect on ERK phosphorylation compared with other compounds, it had the most striking effect on cell proliferation. At concentrations as low as 10 fM, E_2_ increased cell number up to 24% in 3 days, a remarkable sensitivity that has been previously observed for another rat lactotroph cell line (Chun and Gorski 2000). Most of the tested xenoestrogens had no (PP and EP) or little (OP and BPA) effect on cell number (only at the very highest concentrations). The notable exception was NP: 10 pM and above increased cell number 8.5–15% but had no effect on ERK phosphorylation (Figure 5B).

### Hydrophobic properties of xenoestrogen molecules and their rapid effects on cell signaling and function: structure–activity correlations

**Figure 7:**
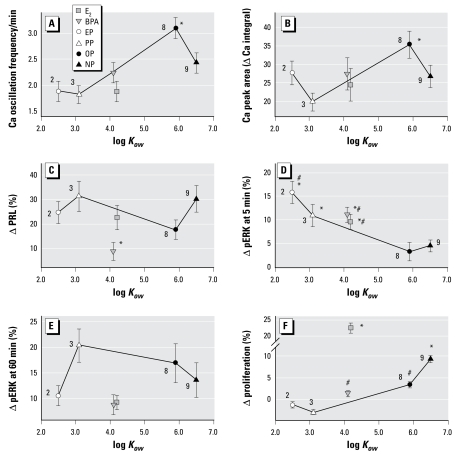
Rapid signaling responses elicited by various alkylphenols, BPA, and E_2_, by increasing hydrophobicity. Responses are ordered by hydrophobicity indices; the numbers next to each symbol are carbon atoms in each compound’s side chain (where applicable). (*A*) Ca oscillation frequency (averaged for 1 fM to 1 nM treatments). *p < 0.05 compared with E_2_, EP, and PP. (*B*) Integral (volume) of the Ca response (averaged for 1 fM to 1 nM treatments). *p < 0.05 compared with E_2_ and PP. (*C*) PRL secretion activated by 100 pM treatment. *p < 0.05 compared with PP and NP. (*D* and *E*) ERK activation response to 1 nM treatments measured at 5 and 60 min, respectively. *p < 0.05 compared with OP; ^#^*p* < 0.05 compared with NP. (*F*) Proliferative response (averaged for 10 pM to 10 nM treatments). *p < 0.05 compared with all other tested compounds; ^#^*p* < 0.05 compared with EP and PP for OP, compared with PP for BPA.

Figure 7 compares all of these rapid cellular signaling and functional responses to structurally related xenoestrogens by increasing hydrophobicity. Hydrophobicity was estimated as the logarithm of octanol–water partition coefficient (log *K*_ow_) taken from the PubChem database (National Center for Biotechnology Information, 2009). Short-chain alkylphenols such as EP and PP have relatively low log *K*_ow_ values, and long-chain OP and NP are relatively highly hydrophobic. E_2_ and BPA, although have no side chains, are similar to each other both in structure (multiple carbon rings) and are intermediate in hydrophobic value. We chose this analysis, compared with the carbon-chain-length comparison used frequently in the literature (Kwack et al. 2002), to formally include compounds without side chains in our characterization. Some simplifications were employed for displaying all these data together, mainly based on limiting concentrations to those causing near-maximal responses. Because most of the Ca responses were dose independent (all-or-none and within these response groups not statistically different from one another), we combined these data to examine the dependence on this chemical feature (Figure 7A, B). For the PRL release data (Figure 7C), 100 pM is shown, and for the proliferative response (Figure 7F), 10 pM to 10 nM treatments were averaged, because they were near maximal for all tested compounds (Figure 4). For the ERK phosphorylation response at both 5 and 60 min (Figure 7D, E), we used the same concentration as for the time courses, because this particular dose consistently produced the most similar biphasic temporal patterns of response for all compounds. This also allowed us to compare responses at two time points for different estrogens.

For producing increases in the Ca firing rate, the more hydrophobic, long-chain alkyl-phenols OP and NP were generally equally active, although OP was significantly better (Figure 7A) than the short-chain EP and PP and extra-ring-structure compounds (BPA and E_2_). Although multiple comparisons tests did not show a statistical difference between NP and the other compounds, it was significantly more effective than EP, PP, and E_2_ by a *t*-test. Only OP was significantly better at increasing the volume of Ca entering cells (Figure 7B). Although the direct correlation between the Ca response parameters and log *K*_ow_ values by Pearson’s test were not statistically significant (*p* = 0.293 for ΔCa integral and *p* = 0.066 for Ca oscillation frequency), by Spearman’s rank correlation test the increase in hydrophobicity from “low” (EP and PP) to “medium” (E_2_ and BPA) to “high” (OP) does significantly correlate with changes in Ca oscillation frequency (*p* = 0.017). No obvious dependence on the chemical properties of the xenoestrogen molecules was seen for rapid PRL release (Figure 7C), except that BPA was significantly less effective than the most effective compounds (PP and NP).

In contrast to the structural dependency of Ca changes, we observed a surprisingly strong inverse correlation between molecule hydrophobicity and the first (5 min) peak of ERK phosphorylation (*p* = 0.004, Pearson test; Figure 7D). No obvious dependence on chemical properties was observed for the second (60 min) ERK phosphorylation peak (Figure 7E). However, E_2_ and BPA again clustered together, being far less effective than would have been predicted by their hydrophobicity scores.

E_2_ and NP were the most effective compounds at causing cells to proliferate (Figure 7F) with a positive correlation with hydrophobicity (*p* = 0.03, Pearson test), and no correlation with their ERK activation profiles at either time point. The magnitude of the E_2_ effect was much larger than its hydrophobicity would have predicted and higher than for any other compound tested.

## Discussion

Our studies demonstrate the abilities of both short- and long-carbon-chain *para*-alkyl phenols, as well as BPA, to potently and effectively cause nongenomic estrogenic responses in pituitary tumor cells. In many of these responses, the xenoestrogens were equivalent to or more potent and effective than E_2_. This clearly shows that these compounds are not “weak” estrogens, as they have been shown to be for many genomic responses (Bonefeld-Jorgensen et al. 2007; Soto et al. 1991; Sumpter and Jobling 1993,^1995^; White et al. 1994).

These xenoestrogens increased the frequency of Ca oscillations and also increased the total cytosolic Ca concentrations in GH_3_/B_6_/F10 somatomammotropes, similar to our previous results for NP and BPA using escalating treatment concentrations on the same cultures (Bulayeva et al. 2005). Our previous studies demonstrated that the increase in Ca influx observed within minutes of E_2_, BPA, and NP application is strictly dependent on mER-α and mediated by L-type voltage-gated Ca channels; EP, PP, and OP likely use a similar cellular machinery to mediate their responses.

Because our GH_3_ cell subline was previously determined not to contain ER-β (Norfleet et al. 1999) or G-protein–coupled receptor 30 (Thomas et al. 2005) under these culturing conditions, these receptors are not likely involved in the responses we describe here. Other closely related receptors that are less well characterized [e.g., the nuclear estrogen-related receptor-γ (ERRγ); Eudy et al. 1998] may participate in Ca and other responses to some xenoestrogens. ERRγ has a much higher affinity for BPA and EP (Liu et al. 2007; Okada et al. 2008) and could contribute to these results. The possibility of different receptor involvement could explain other observations by Woclawek-Potocka et al. (2006) that EP did not affect Ca signaling in the endometrium but did affect prostaglandin F_2__α_ production, whereas E_2_ affected both.

A relatively long (several minutes) and highly variable delay between the application of the estrogens and their observed effects on increasing the Ca oscillation frequency, as well as the lack of a clear concentration dependence of the response magnitudes, suggests that the mechanism of estrogen and xenoestrogen action on Ca signaling involves multiple intracellular events. These probably include phosphorylation of multiple signaling proteins and their targets by numerous different kinases, and perhaps also changes in membrane excitability. The fact that only spontaneously active cells (generating episodic Ca waves without any stimulation) typically responded to these estrogens indicates indirectly that E_2_ and these xenoestrogens may elicit their actions by changing resting potentials. Because a major mechanism for regulation of membrane excitability in pituitary lactotropes is potassium channel modulation caused by TRH (Bauer 1998), crosstalk is likely with its signaling pathways for inducing these estrogen/xenoestrogen-mediated Ca responses. One interesting direction for further studies would be a more thorough examination of the possible crosstalk between multiple signaling pathways activated by E_2_ and xenoestrogens, as suggested by our previous work (Bulayeva et al. 2004, 2005; Wozniak et al. 2005).

Despite the relatively long lag time for estrogen-induced Ca firing changes, the rapid effect on PRL release is completed within 1 min. These data suggest that besides activation of Ca signaling, estrogens regulate PRL release through additional rapid mechanisms, probably related to secretory granule maturation, and docking and priming, as we demonstrated previously by showing that very large Ca effects do not necessarily cause very large secretory responses (Bulayeva et al. 2005). Further study of these possible mechanisms would contribute to our understanding of estrogen- and xenoestrogen-dependent regulation of endocrine functions. In addition, limitations of the fura-2 technique undoubtedly contribute to our inability to precisely detect fast and local Ca transients in these experiments. Further studies employing membrane Ca-current patch-clamp recording and advanced microscopic techniques that allow us to observe the near-membrane Ca domain could provide further details of how estrogens activate Ca signaling events, which in turn influences quantal PRL release.

The functional consequences of activating the ERK cascade in these cells cannot easily be related to cell proliferation as in some other systems (Meloche and Pouyssegur 2007). Overall, the biphasic time courses of ERK responses were similar between these alkylphenols and E_2_, suggesting common signaling pathways initiated at the mER-α. Other classes of xenoestrogens (the chlorinated pesticides dichlorodiphenyldichloro-ethylene, dieldrin, and endosulfan) that we previously studied elicited very different time courses of ERK activation and were differentially dependent upon upstream kinases at different times (Bulayeva and Watson 2004). With the exception of EP, these compounds showed an inverse correlation between the magnitude of their usually proliferation-related ERK activation at 60 min (PP > OP > NP > E_2_ > BPA) and their binding affinity for the artificially expressed nuclear receptor reported in the literature (Bonefeld-Jorgensen et al. 2001; Kwack et al. 2002; Routledge and Sumpter 1997). Therefore, the rules for potency and effectiveness via the mER-α versus the nuclear ER appear to be different. These data may suggest that the effects of estrogens on pituitary tumor cell proliferation are realized via distinct or additional pathways. Our other recent data suggest additional pathways, because blocking estrogenic activation of ERKs and other mitogen-activated protein kinases blocks cell proliferation in these cells (Jeng Y-J and Watson CS, unpublished data). Assessment of changes in mitotic activity, apoptosis, cell attachment, and motility may further our understanding of these differential estrogenic effects on this cell type’s ability to proliferate. Properties of these molecules other than hydrophobicity may affect their ability to activate ERK signaling, and the functional consequences of ERK signaling may not include or extend beyond cell proliferation.

Multiple aspects of rapid mER-mediated signaling in pituitary cells do not consistently correlate with the affinity of xenoestrogen molecules to the artificially produced nuclear form of this receptor (Bonefeld-Jorgensen et al. 2007; Kwack et al. 2002; Routledge and Sumpter 1997). In some cases we even observed a reverse dependence, as in the case of the delayed (60 min) ERK activation. As is evident from our dose–response curves describing effects of xenoestrogens on Ca signaling and PRL release, the active concentrations needed to produce a response (100 fM to 1 nM) were far below those reported in the literature for alkylphenol and BPA genomic responses. These concentrations are also far lower than those reportedly required for half-maximal binding to heterologously expressed and/or purified ER reporter gene activation. This difference is likely explained by distinct ligand-binding properties of mER-α due to structural differences (posttranslational modifications; Li et al. 2003) or a dramatic impact of the microenvironment (membrane border lipid contact). This emphasizes the importance of further structural/functional studies specifically on mER-α, because its ligand interactions appear to be dramatically different from those of the cytosolic/nuclear receptor.

Structure-based activity models of chemical endocrine disruptors would be very helpful for safety screening and predictions of exposure outcomes (Devillers et al. 2006). This might be particularly important for membrane receptor activation, because the rules affecting receptor affinity and activity may be different and are as yet little explored. Our data suggest that the ability of xenoestrogens to elicit rapid Ca and ERK phosphorylation responses in pituitary cells depends on hydrophobicity, which is somewhat dependent upon the length of linear carbon chains, rather than with their ability to bind nuclear receptor in artificial binding systems. The Ca and ERK responses correlate with hydrophobicity in the opposite way: the more hydrophobic the compound, the bigger the Ca changes but the less ERK phosphorylation produced. This may suggest that different conformational states of mER-α caused by the docking of differently shaped ligands, as well as their induced membrane microenvironment changes, might dictate different interactions with other signaling molecules and thus different biologic response propensities.

## Figures and Tables

**Figure 1:**
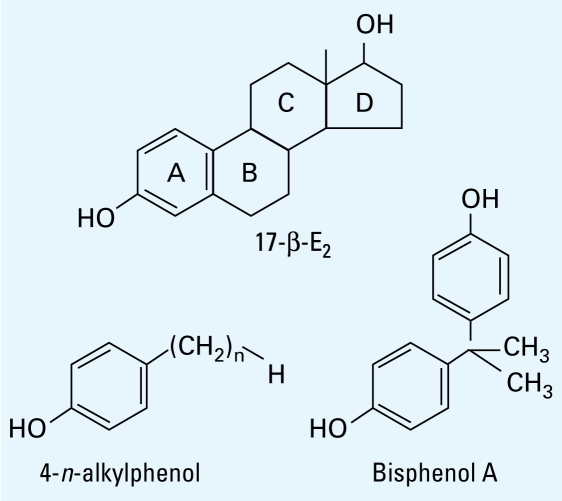
Structural similarities among E_2_, BPA, and *para*-substituted phenolic compounds [(CH_2_)*_n_*; *n* = 2, 3, 8, and 9 for the alkyl-phenols in our studies] with possible estrogenic activities. The phenol backbone of BPA and alkylphenols show structural similarity with the hydroxylated phenolic A-ring of E_2_, which appears to be essential for ER binding (Tabira et al. 1999).
